# Barriers and Facilitators to Behavioral Healthcare for Women Veterans: a Mixed-Methods Analysis of the Current Landscape

**DOI:** 10.1007/s11414-023-09862-3

**Published:** 2023-10-05

**Authors:** Reagan E. Fitzke, Kathryn E. Bouskill, Angeles Sedano, Denise D. Tran, Shaddy K. Saba, Keegan Buch, Justin F. Hummer, Jordan P. Davis, Eric R. Pedersen

**Affiliations:** 1 Department of Psychiatry and Behavioral Sciences, University of Southern California Keck School of Medicine, 2250 Alcazar St., Suite 2200, Los Angeles, CA 90089 USA; 2 RAND Corporation, 1776 Main St, Santa Monica, CA 90401 USA; 3 University of Southern California, Suzanne Dworak-Peck School of Social Work, 669 W. 34th St, Los Angeles, CA 90089 USA

## Abstract

Women veterans have historically faced barriers to behavioral health treatment, particularly through the VA. In conjunction, there have been changes in behavioral healthcare delivery resulting from efforts to improve care for women veterans and the COVID-19 pandemic (e.g., widespread telehealth implementation). The current study draws on a quantitative and qualitative study centering current perspectives of women veterans in their choices to seek or not seek behavioral healthcare in VA and non-VA settings through interviewing 18 women recruited from a larger survey study on veteran behavioral health (*n* = 83 women, *n* = 882 men) on their experiences with behavioral health care access and satisfaction, including barriers and facilitators to seeking care. Quantitative findings are descriptively reported from the larger study, which outlined screening for behavioral health problems, behavioral health utilization, treatment modality preferences, and barriers/facilitators to care. While women in the survey sample screened for various behavioral health disorders, rates of treatment seeking remained relatively low. Women reported positive and negative experiences with telehealth and endorsed many barriers to treatment seeking in interviews not captured by survey findings, including lack of women-specific care (e.g., care for military sexual trauma, women-only groups), reports of stranger harassment at the VA, and lack of female providers. Women veterans continue to face barriers to behavioral healthcare; however, ongoing efforts to improve care access and quality, including the implementation of telehealth, show promise in reducing these obstacles. Continued efforts are needed to ensure diverse treatment modalities continue to reach women veterans as this population grows.

## Introduction

US veterans have a high prevalence of behavioral health concerns, with PTSD, depression, anxiety, and substance use disorders being among the most commonly diagnosed.^1^ Despite this, many do not seek treatment.^2, 3^ For example, nearly 1.3 million US veterans have a substance use disorder (SUD), most commonly alcohol use disorder (AUD), but upwards of 85% of veterans with SUDs do not seek SUD-related care.^1^ Veterans endorse barriers to seeking treatment, including negative beliefs about care, perceived stigma surrounding mental health, and a military culture of self-reliance (e.g., “pulling yourself up by the bootstraps”).^4^ Continual investigation of barriers to behavioral healthcare for veterans is important to guide ongoing efforts to increase care utilization, especially for subgroups who may have unique behavioral health needs, such as women veterans.

### Behavioral healthcare for women veterans

Demographics of veteran populations are shifting: 12% of US veterans were women in 2020, compared to 4% in 2010; this may reach 18% by 2040.^5–7^ Importantly, women veterans face unique behavioral health-related challenges.^8^ Relative to their male veteran counterparts, women veterans are more likely to be diagnosed with depression (23–48% vs. 17–32% across studies) and anxiety (12–20% vs. 10–16% across studies).^9–11^ Furthermore, women veterans may have more frequent co-occurring behavioral health problems than men.^11^ In one study, although women veterans were less likely to screen for AUD, women with AUD were nearly twice as likely to have concurrent mental health diagnoses (e.g., depression, PTSD, anxiety) than veteran men with AUD.^12^

While healthcare utilization among women veterans is increasing overall,^13^ behavioral healthcare utilization is still lagging among certain subgroups, including rural or racial and ethnic minority women.^14^ Reports of care dissatisfaction also warrant concern such that even when women veterans present for care, providers may not be meeting their needs.^14^

A recent systematic review highlighted the pressing need for research to better understand barriers faced by women veterans in accessing women-focused behavioral health treatments.^4^ First, women veterans experience disproportionately high rates of military sexual trauma (MST)^15^ in comparison to men; though there are routine screenings and other care available for MST within Veterans Affairs (VA) centers, there remain barriers to seeing this care.^16^ For instance, women veterans can experience stranger harassment at primarily male-dominated clinics,^17^ which may discourage initial treatment pursuit or continued care. Women also report barriers to disclosure of MST, such as fear of retaliation and shame.^15^ Moreover, post-9/11 women veterans are also more likely than older women veterans to have combat experience.^18^ Thus, women have likely had increasing exposure to traumatic experiences that could compound with their unique challenges and lead to further behavioral health concerns. There is thus a continued need to increase access and dismantle barriers to behavioral health resources for women veterans.^8^

The VA has taken initial strides to improve healthcare for women veterans, beginning with the 2004 Women’s Health Research Agenda, which bolstered VA research efforts to improve healthcare access and quality for women veterans.^19^ For example, this initiative has included adaptations aimed at eliminating disparities for women’s mental health (e.g., targeting interventions for higher suicide, partner violence rates among women), with continued expansion upon this agenda to meet this population’s growing care needs.^20^ Despite the VA’s efforts to identify and implement strategies to circumvent women veterans’ barriers to care, estimates still indicate only 22% of women veterans use VA services.^21^

### Current behavioral healthcare considerations

The COVID-19 pandemic exacerbated behavioral health symptoms among veterans with pre-existing conditions^22^ and simultaneously drove the growth of telehealth as a relatively common behavioral health treatment modality within the VA.^23–25^ Concurrently, there have been efforts to adapt interventions to telehealth during the pandemic for women veterans.^26^ Despite these efforts, women veterans faced ongoing challenges in getting their behavioral health needs met throughout the pandemic,^27, 28^ consistent with pre-pandemic trends.

The behavioral health needs of women veterans are wide-ranging and often unmet, and the factors affecting care utilization are likewise complex and dynamic. The impact of telehealth implementation during COVID-19 is understudied, particularly in the context of ongoing attempts to reduce barriers to care for women veterans. Furthermore, little is known about the barriers and facilitators of care for women veterans outside of VA care settings, or how veteran women and men differ in their experience of such barriers and facilitators. The current study thus endeavored to gather unique and nuanced insights into these factors among a sample of post-9/11 women veterans who are diverse in their utilization of VA/non-VA care, as well as among those who may not report seeking any behavioral healthcare. This study used both quantitative data from self-report survey methods and qualitative data derived from individual interviews. This study began with data from a nationwide survey that assessed veteran behavioral health broadly, which included established survey measures on behavioral health symptomology and treatment attitudes (e.g., barriers and facilitators). Knowing that survey measures were developed with majority male samples, and because our study aimed to specifically understand the experiences of women veterans, the current study compared women’s responses to those of the veteran men in our sample, and contextualized quantitative findings with qualitative data specific to women veterans’ experiences.^29^

## Methods

### Participants and procedures

The University of Southern California Institutional Review Board approved all study procedures. Participants were recruited from general and veteran-targeted social media websites (Facebook, Instagram, RallyPoint, We Are The Mighty) in February 2020 for a larger study on veteran behavioral health; for details, see Pedersen et al.^22^ Social media was the primary recruitment method to target veterans recruited outside of additional VA settings. US veterans from the Navy, Air Force, Marine Corps, and Army between the ages of 18 and 40 were eligible. Veterans could also not be currently affiliated with the military via active duty service or in the reserve or guard units. Veterans did not need to have any behavioral health disorders or treatment experience at the VA or outside the VA to participate. A total of 1855 veteran participants were initially enrolled. Efforts to minimize fraudulent participation by computer bots or non-veteran individuals attempting to complete the survey were implemented, including removing participants that failed internal validation checks. The final sample size was *N* = 1230. To examine veterans’ behavioral health during COVID-19, follow-up surveys were sent at 6 months (August 2020; *n* = 1025; 83.3% retention), 9 months (November 2020; *n* = 1006; 81.8% retention), 12 months (February 2021; *n* = 1005; 81.7% retention), and 18 months (August 2021; *n* = 967; 78.6% retention) post-baseline. Participants received a $20 gift card for completing each of the baseline and 18-month follow-up surveys. The analytic sample for the current study includes demographic data from the baseline survey and the 18-month follow-up survey. As very few participants reported a gender identity that differed from their sex at birth (*n* = 2), this study only includes cisgender women (*n* = 83) and men (*n* = 882) in the final analytic sample for statistical power and generalizability purposes (*n* = 965).

After the first follow-up survey in August 2020, a subset of participants completed a 60-min qualitative interview via Zoom to discuss how veterans were coping with COVID-19. Twenty-three participants completed initial interviews. Detailed methods and findings from initial interviews are detailed in other published work.^30^ Women from the initial interviews were invited for a follow-up interview after completion of the 18-month survey in Fall 2021. Five of the seven women participants who completed the first interview agreed to an additional interview. Seeking to learn more about women veterans’ unique experiences related to behavioral health care, especially due to limited women-focused veteran behavioral health research amid COVID-19, the authors recruited and enrolled an additional 13 women veteran participants by emailing an additional subset of women from the larger study to inquire about interest in completing an additional paid interview with the study team. Thus, 18 women veterans total (22% of the study sample) completed the qualitative interview for the current study, which is generally a sufficient number of participants to reach thematic saturation.^31^ Participants were compensated with a $50 gift card for completing each interview and a $30 gift card for each follow-up survey. See Table 1 for demographics of the 18 interview participants, and men (*n* = 882) and women (*n* = 83) participants who completed the 18-month follow-up survey. This study reports methods and findings from questions unique to the 18-month follow-up interview.

**Table 1:** Analytic sample (men/women) and interviewee (women only) demographics

		Men (n = 882)		Women (n = 83)		Interview sample (n = 18)	
		Mean	SD	Mean	SD	Mean	SD
Age		34.6	3.4	32.8	4.9	34.0	3.6
Years served		10.2	3.3	6.7	3.9	7.3	4.3
		*N*	%	*N*	%	*N*	%
Branch of service							
	*Air Force*	96	10.9%	19	22.9%	1	5.6%
	*Army*	640	72.6%	41	49.4%	13	72.2%
	*Marine Corps*	84	9.5%	11	13.3%	3	16.7%
	*Navy*	12	14.5%	12	14.5%	1	5.6%
Race and ethnicity							
	*Hispanic or Latino*^*1*^	66	7.5%	16	19.3%	4	22.2%
	*Black*	41	4.6%	7	8.4%	0	-
	*Asian*	5	0.6%	0	-	0	-
	*White*	761	86.3%	60	72.3%	14	77.8%
	*Multiracial/Not listed*	9	1.1%	0	-	0	-
Mental health screening							
	*Depression screen*^*2*^	309	35.0%	39	47.0%	10	55.6%
	*Anxiety screen*	199	22.6%	38	45.8%	11	61.1%
	*PTSD screen*^*3*^	172	19.5%	36	43.4%	9	50.0%
	*Hazardous drinking screen*^*4*^	745	84.5%	26	31.3%	2	11.1%
	*Alcohol use disorder screen*^*4*^	197	22.3%	13	15.7%	0	-

^1^Hispanic/Latino ethnicity was assessed separately from race

^2^Participants screened for probable depression at summed scores higher than 10 on the Patient Health Questionnaire-8 (PHQ-8)

^3^Participants screened for probable anxiety at summed scores higher than 10 on the Generalized Anxiety Disorder-7 (GAD-7)

^4^Participants screened for hazardous drinking if they scored a sum of 8 or above, and for probable alcohol use disorder with a score of 16 or above, on the Alcohol Use Disorders Identification Test (AUDIT)

### Survey measures

Demographics were assessed at baseline, and measures of behavioral health, behavioral healthcare usage and satisfaction, and barriers and facilitators of treatment were assessed at the 18-month follow-up survey.

### Demographics

Participants reported on age, race and ethnicity, branch of service, and years served in the military.

### Behavioral health screening measures

#### Depression

Depression was measured with the 8-item Patient Health Questionnaire,^32^ which assessed specific depression symptoms (e.g., feeling down, depressed, and hopeless) over the past 2 weeks. Participants rated each item from “not at all” (0) to “nearly every day” (4). A summed score of 10 or more was used as the cutoff for a positive depression screen.^32^

#### PTSD

PTSD symptom severity was assessed using the 20-item Posttraumatic Stress Disorder Checklist for DSM-5 (PCL-5).^33, 34^ Participants indicated how often they were bothered by 20 symptoms of PTSD in the past month (e.g., hyperarousal, unwanted memories of a traumatic event) from “not at all” (0) to “extremely” (4). A summed score of 33 or more indicated a positive PTSD screen.^33^

#### Anxiety

Anxiety was assessed using the Generalized Anxiety Disorder 7-item questionnaire (GAD-7). Items assessed how often participants were bothered by anxiety symptoms (e.g., feeling nervous, anxious, on edge; excessive worrying) in the past 2 weeks. Participants rated each item from 0 = not at all to 3 = nearly every day, with a cutoff sum score of 10 indicating probable anxiety disorder.^35^

#### Alcohol use disorder

Participants completed the 10-item Alcohol Use Disorder Identification Test (AUDIT).^36^ The AUDIT assesses the frequency of symptoms of alcohol use disorder in the past year (e.g., failing to do what was normally expected due to drinking, not able to stop drinking once started). A cutoff score of 8 indicated a positive screen for “hazardous drinking,” while a score of 16 indicated a positive screen for alcohol use disorder.^37^

### Behavioral healthcare usage and satisfaction

Participants reported past-6-month behavioral healthcare treatment utilization (defined as appointments for concerns relating to mental health or substance use, including outpatient psychotherapy, inpatient care, medication, and peer support groups). Participants indicated whether or not they received care through (a) VA and/or non-VA services, or via (b) in-person and/or telehealth in the past 6 months at the time of the survey (February 2021–August 2021). If participants endorsed receiving any modality of care, a series of follow-up questions asked about treatment frequency (days in past 6 months), separately for each provider and modality combination (e.g., “How many days in the past 6 months have you attended appointments [IN PERSON/ONLINE] at [the VA/a non-VA provider] to help you with a mental health or substance use concern?”) for all treatments endorsed. Those who received telehealth care were also asked to compare perceived quality of care for in-person vs. telehealth services (i.e., in-person better, telehealth better, both the same, or did not know/could not compare [due to never receiving in-person care]), separately for VA and non-VA services.

### Perceived barriers and facilitators to treatment

**Fig. 1:**
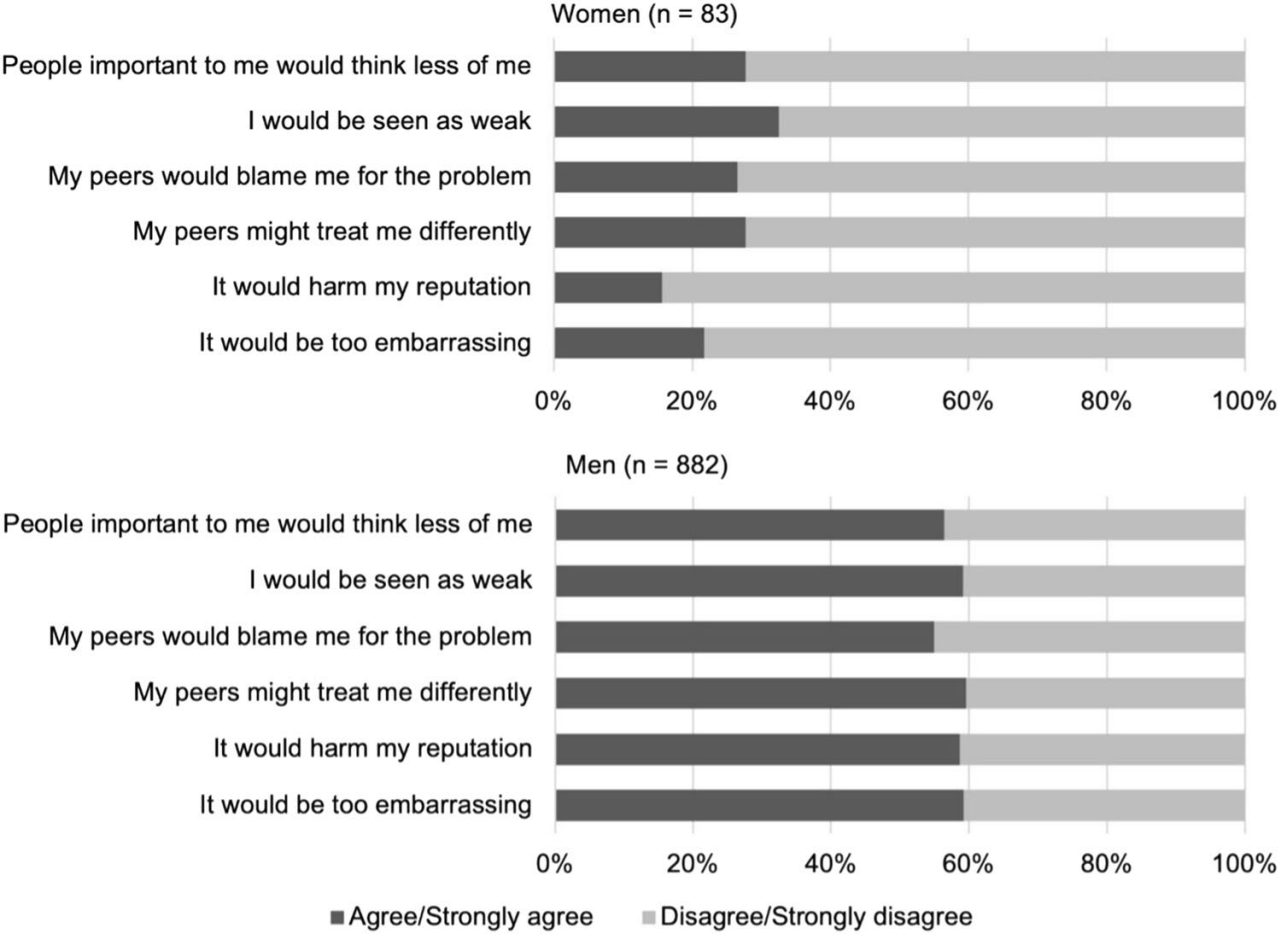
Percent endorsement of barriers to behavioral health care questionnaire. *Note*. Strongly agree/agree and strongly disagree/disagree responses were combined for the barriers to aid interpretation

**Fig. 2:**
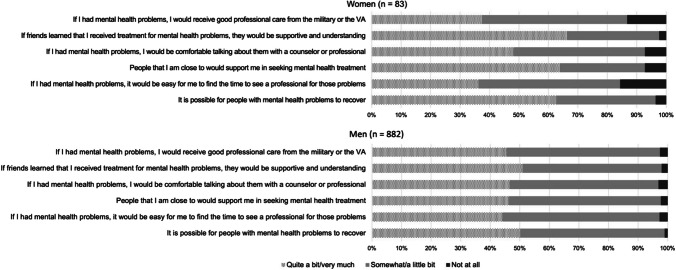
Percent endorsement of facilitators to behavioral health care questionnaire. *Note*. Response options of very much/quite a bit and somewhat/a little bit are presented together in the figure to aid interpretation of results

Barriers to receiving behavioral health treatment were assessed with the Perceived Stigma and Barriers to Care for Psychological Problems scale, which was developed for use with young adult service members and veterans.^38, 39^ Items generally assessed perceived stigma from others and asked how each of the six items might affect one’s decision to seek treatment for a psychological problem from a mental health professional (e.g., a psychologist or counselor). Items were rated from “strongly disagree” (1) to “strongly agree” (4). For facilitators of care, participants completed the 6-item RAND Facilitators of Mental Health Care questionnaire,^40^ which assessed facilitators of receiving care from a behavioral health provider. Items were rated from “not at all” (1) to “very much” (5). Means for both 6-item scales were computed as overall scores for barriers (higher mean is higher agreement with barriers) and facilitators (higher mean is greater endorsement of facilitators). Individual items for the barriers and facilitators scales are listed in Figs. 1 and 2.

### Qualitative interview protocol

Semi-structured interviews were initially designed to serve either (1) as a follow-up from a round of interviews conducted in 2020 or (2) as an initial interview, which both assessed the same past-year timeframe.^30^ The purposes of these interviews were to further inform and contextualize quantitative findings from the larger study. Interviews followed a standardized guide and included retrospective questions on behavioral health changes during the COVID-19 pandemic among veterans. Interviews did not specifically probe about behavioral health diagnoses, but rather centered on health, well-being, and access to treatment during COVID-19. In addition, interviewers asked questions regarding women’s perspectives on behavioral healthcare access for women veterans broadly (i.e., both during and prior to the pandemic). Interviews further probed about specific positive and negative experiences with VA care and asked if participants had any further thoughts about how women veterans could be better served.

### Analysis plan

#### Survey data

Descriptive statistics on sample demographics, behavioral health screening, treatment utilization, and perceived barriers/facilitators to behavioral healthcare among men and women in the sample are reported. To assess differences in treatment utilization, this study conducted *Z*-tests to compare the proportion of men and women in the sample who endorsed VA or non-VA treatment in the past 6 months at the time of the 18-month survey. Due to sample size, this study lacked the statistical power to test for differences between smaller treatment subgroups (in-person vs. online within the VA and non-VA). Furthermore, *t*-tests assessed for mean differences on reported barriers and facilitators of care between men and women.

#### Interview data

Interviews were recorded following participants’ consent, transcribed, and uploaded into Dedoose.^41^ The research team created a codebook based on the interview protocol and key research questions. The coding team, which consisted of three graduate-level research assistants supervised by a doctoral level anthropologist, first co-coded two interviews and then refined the codebook and application of codes. The coding team met regularly to discuss any responses that were unclear. After 20% of the interviews were coded, interrater reliability was calculated with a pooled Cohen Kappa coefficient and Cohen Kappa for each of the codes.^42^ Coding procedures were refined until the pooled Cohen Kappa coefficient and Cohen Kappa for each code was > 0.80, which demonstrates a high level of agreement and consistency across the qualitative analytic team^42^ and is accepted as a strong level of agreement among coders. Coding was followed by thematic analysis.^43, 44^ The authors followed standard approaches to identify key themes, or the range of responses under each code, by noting specific words, phrases, and ideas.^43^ The study team identified additional themes through repetition, metaphors used, and existing literature of sources of veterans’ healthcare-related perceptions.^44^

## Results

Results are first presented organized by quantitative survey findings and are then supplemented and contextualized by qualitative narrative accounts, following a well-established mixed methodology approach.^45^ This allows for a more nuanced understanding of behavioral health treatment seeking among post-9/11 women veterans and areas for improvement in facilitating care.

### Behavioral health problem prevalence

See Table 1 for proportions of women and men from the larger survey study and interview subsample who screened for behavioral health problems. Overall, high rates of probable depression and PTSD were observed among the full sample, though a significantly higher percentage of women screened for probable depression (47% vs. 35%; *X*^2^ [1, 966] = 5.16, *p* = 0.023), PTSD (43.4% vs. 19.5%; *X*^2^ [1, 966] = 26.91, *p* < 0.001), and anxiety (45.8% vs. 22.6%; *X*^2^ [1, 966] = 23.24, *p* < 0.001). A significantly higher percentage of men screened for hazardous drinking (85% vs. 31%; *X*^2^ [1, 967] = 131.03, *p* < 0.001), but men and women reported comparable rates of AUD (22% vs. 16%). Women in the interview sample screened positive for depression, anxiety, and PTSD more frequently than in the full sample, and hazardous drinking or AUD less frequently than in the full sample. Overlaps between positive screens for disorders were typical for both women and men, with 78.2% of the women in the survey sample who screened positive for at least one of the four disorders (depression, PTSD, anxiety, AUD) screening positive for more than one disorder, 86.7% of women who screened positive for at least one disorder in the interview sample screened positive for more than one disorder, and 39.2% of men who screened positive for at least one of the four disorders in the survey sample screened positive for more than one disorder.

### Behavioral healthcare usage and satisfaction

#### Survey findings

Treatment usage among the analytic sample is described in Table 2. The proportion of women utilizing behavioral healthcare during the defined period was significantly higher than the proportion of men utilizing care for both VA (13.3% vs 4.5%; *Z* =  − 3.39, *p* < 0.001) and non-VA (4.3% vs 9.6%; *Z* =  − 2.18, *p* = 0.03) services. Overall, treatment utilization was split across systems of care (VA vs. non-VA) and administration (in-person vs. telehealth vs. both). Of the women who received telehealth care from the VA, 27.8% indicated in-person care quality was better, 38.9% reported favoring telehealth, and 33.4% reported care was the same quality or could not compare. For non-VA services, 26.4% of women reported in-person care quality was better; 31.6% believed telehealth was better; 42.2% stated care was the same quality/could not compare. Overall, it did appear that women and men in the sample who reported receiving treatment in the past 6 months were those who screened positive for at least one of the behavioral health disorders assessed (depression, PTSD, anxiety, AUD). Seventy-five percent of women in the sample and 90% of men in the sample who reported receiving treatment also screened positive on the survey for one of the assessed disorders. However, of those who screened positive for at least a disorder in the survey sample, 55% of women and 43% of men who screened positive reported not receiving any behavioral health care in the past 6 months.

**Table 2:** Behavioral health care receipt (February–August 2021) based on location (VA vs. non-VA) and type of care (in-person vs. online)

		Women (n = 83)		Men (n = 882)		Z (p)
Type of care received		N (%)	M (SD) days in past 180	N (%)	M (SD) days in past 180	
VA behavioral healthcare use in past 6 months						− 3.71 (< 0.001)*
	No	45 (54.2%)		732 (83.0%)		
	Yes^1^	38 (45.8%)		150 (17.0%)		
	In-person	22 (57.8%)	3.91 (4.07)	100 (66.7%)	3.25 (3.09)	
	Telehealth	27 (71.1%)	4.26 (5.22)	90 (60.0%)	2.04 (2.13)	
Non-VA behavioral healthcare use in past 6 months						− 2.61 (0.009)*
	No	50 (60.2%)		759 (86.1%)		
	Yes^1^	33 (39.8%)		123 (13.9%)		
	In-person	20 (60.6%)	7.05 (11.25)	84 (68.3%)	3.12 (3.01)	
	Telehealth	21 (63.6%)	2.67 (3.24)	77 (62.6%)	3.86 (6.38)	

^*^Differences in proportion of men vs. women endorsing treatment utilization significant at *p* < *0.05*

*Note. Z*-tests were only conducted on overall engagement of VA and non-VA behavioral healthcare due to low *N* of tx subtypes

^1^Percent of in-person vs. telehealth care receipt was calculated out of those who indicated any behavioral health care receipt of the specified VA vs. non-VA type in past 6 months

#### Interview findings

Key qualitative findings are summarized in Table 3. Women recalled experiences of VA and non-VA behavioral healthcare access and receipt as applicable. Some women described not using VA care services due to past negative experiences, while others reported recently seeking care through the VA. Women also reported using VA telehealth services during the pandemic. Women expressed satisfaction with the quality and ease of access to treatment with technology provided by the VA (e.g., apps, tablets). They also expressed telehealth easing barriers to seeking care within the VA; for example, one participant described telehealth easing concerns about talking to staff and providers due to her social anxiety. Others indicated difficulty with connecting to services virtually or feeling in-person treatment would better meet their behavioral health needs (e.g., building rapport, noticing non-verbal cues).

**Table 3:** Key findings from qualitative results surrounding women’s behavioral health and care receipt experiences

Key themes and sub-themes	Exemplary quote
Telehealth access during COVID-19	“I got an iPad from the VA…because I had the social anxiety and didn’t want to go out and see the team there. It made it easier… My only issue is when it started, what was making me paranoid is when they wanted me to do group stuff because of the social anxiety. I always have a problem with everything I say, like I go back through my head and I’m like, ‘That was stupid. Why did you say that?’ …That’s intensified when there’s multiple people.” “Now [the VA] has their own app that you set your appointments and then they’ll email you or text you a link for that specific appointment. And then when the time comes a few minutes early, you can log in to get everything set up. And then it’s pretty much like a Zoom appointment… You talk and have that appointment over the phone like that. Or you can do it just voice and that’s how I do my appointments now. So, I talked to [my provider] once a week doing that. … I had [a provider] before. But he never actually spoke with me about the mental health issues. It was just more of, ‘Hey, do you feel better?’ I was like, ‘no’…because they’re not addressing anything, they’re not doing any therapy sessions or anything. He was just like, ‘Do you feel better? I’ll go ahead and double your medication,’ and to the point where I wound up getting sick and that’s why I had to stop it. And then [a provider] I had that didn’t believe anything that I had said… That was my first one actually right after… because I was assaulted in the military…So, it took eight years for me to finally find the one I have now. And she’s amazing.” “I like online convenience as far as being able to be wherever I am at and getting it done, but that’s about it. Otherwise, I don’t like it, I really don’t... the thought that I just had too is like, I can hide symptoms more.”
Women-specific barriers to healthcare	
Being part of a minority within veterans as a woman	“Some of the mental health stuff that I went to, I was the only woman in there, which was an interesting experience because our experiences, I think sometimes are different than men’s. But that’s probably about it, just the numbers…there’s just not as many women veterans as there are men.”
Needs for women not being met within treatment	“I left [inpatient treatment] against medical advice because I told myself that if that’s the way they were going to treat me, I could treat myself better at home and just sit at the kitchen table… At that second hospitalization, I wasn’t allowed, they didn’t give me pads or tampons, even though I started my period, I had to wash my underwear in the sink. I wasn’t given even a sports bra to wear. And I’m a bustier girl. I got to the point where I was like, what day is it? And they told me, and I was like, I have to leave. My son has a doctor’s appointment, and it took us two years to get this appointment. I’m not going to forego it because you guys are telling me I need to talk about things and get over my trauma.”
Discrimination against women at the VA	“Being a female vet walking in there, you know, I would get eyeballed. ‘*You’re a female, you don’t belong here.*’ You know, there was a lot of underhanded comments from the old school vets, Vietnam era. ‘*You weren’t on the front lines; you were a paper pusher*.’ Well, I’m sorry, but what they don’t know is I was actually a convoy gunner and yes, I was out there in the middle of the desert and manning my machine gun in a Humvee. I saw a lot more than most people did, and it still haunts me to this day.”
Lack of women-specific care	“I got lucky because I have a VA [where] they have full women’s health clinic. But even with that, you know, the amount (sic) of female veterans in a Metro area, there’s really still not enough medical help for all of them. It seems like the doctors are overbooking at times. They have…a very large caseload of patients. They need to bring in more women doctors and they need to start bringing in women vets to the mix to tell them, you know, this is what we need.”
Military sexual trauma	
*MST being common among women in the service*	“I can tell you that didn’t ever happen to me personally, but sexual assault is a very big thing… In the military in general, one of my battle buddies, she experienced it from one of our NCOs. And I only learned about that four years ago … and that sucks because like, I know exactly when it happened, when she started telling me what happened. I knew I wasn’t there, but I knew something had to happen because a couple of guys from our unit had brought her to my barracks room and like I was intoxicated and in my own little world. And so like, when you’re there, you don’t recognize what’s happening, but looking back, like, I don’t know. So that sucks. ‘Cause I wish… that she would have felt more comfortable telling me back then because I would have beat everybody up for her, you know?”
*Stigma surrounding reporting MST*	“Quit looking at us like we’re just trying to get a paycheck whenever we report military sexual trauma. Yeah. I’m tired of that. Yeah. I’m extremely tired of that. When I reported my case, I was threatened by CID to retract my complaint, or I would go to Fort Leavenworth for adultery. I’m sorry. I did not have consensual sex with him. I was raped. I think if society would be more supportive towards military veterans and sexual assaults… I think that if we had society behind us that maybe we could eradicate it.”
*Need for MST-specific care*	“I was… raped in the military, so they need to have more females [at the VA] and [providers] need to be a little bit more trained on that. And then just the combat PTSD, because a lot of us dealt through military sexual trauma, but the PTSD that they’re more focused on is a combat-related one.”
Sexism in military culture	“Go in expecting to be put down, go in expecting to make, go in expecting to have to prove your worth to every man that you come across. But when do you prove your worth, it will be so worth it to get that real level of respect. Go [into the military], knowing that you will have to put in a lot of hard work to get to where you need to go. Be prepared.” “A woman’s experience depends on her command. Just as much as her experience when she gets out, depends on the same kind of premise her care team… We say we have equality, but we don’t, I guess that’s all there is to say.”
Healthcare facilitators	
Positive changes in VA	“[VA providers] have women’s therapy clinics, you know, specifically for my type of trauma that I’ve had. They actually have it like therapy sessions or groups, which are specifically just for females, if they’re not comfortable being around males. Which is amazing. Because I actually, I had done one of those myself, and the different types of therapies available and care… it seems like it’s building up. Like it’s been getting better over the past few years…. I mean there’s always room for improvement, but better from when I got out in 2015. It’s a big difference. Like it’s a lot more helpful and I feel like we’re actually cared for now more, and not seen as you know, “are you here for your husband or are you here for, you know…” I’ve not seen that much anymore and I’m seeing a lot more female veterans going in at the VA as opposed to when I first started going to the VA.”
More options for women enhance ease of care access	“I don’t know if any of it is because I’m a woman… For my primary care, because I go through women’s health, I feel like it’s probably easier to get an appointment.” “Access to childcare [would be helpful]. Especially when we’ve got to go to two appointments, maybe having childcare options or something like that.”
Wanting equal treatment	“I don’t feel that veterans should be treated differently, male or female. And I don’t see like personally where any portion of my life would differ from a male. I mean, like I said, other than literally a gynecologist appointment, I just don’t feel that women nor men should be deviated that way in the veteran community. I didn’t join as a woman veteran. I joined as a veteran. I feel like we put ourselves on an equal playing field regardless of gender, creed, color when we enlist. And I don’t feel that that should change just because our enlistment’s over.”

### Barriers to behavioral health treatment

#### Survey findings

Barriers to treatment reported by the sample are reported in Fig. 1. Overall, fewer women endorsed barriers to treatment seeking than men in the survey (*M*_women_ = 2.08, *SD* = 1.13; *M*_men_ = 3.33, *SD* = 0.85; *t*(962) = 9.83, *p* < 0.001). Most men in the sample agreed or strongly agreed with all statements regarding barriers, while most women either strongly disagreed or disagreed with such statements.

#### Interview findings

Women described challenges to seeking treatment for PTSD symptoms related to experiences of MST, many of which described seeking treatment through VA providers. They reported providers were unable to understand their experiences sufficiently, or that group settings were particularly difficult to engage in, as many other veterans only discussed combat-related trauma. Other barriers specific to women included feeling judged or dismissed by VA healthcare providers and other veterans in the clinic, such as providers not taking their prior military service seriously or being harassed by veteran men in the waiting room before an appointment, and a lack of female providers in some locations.

### Facilitators to behavioral health treatment

#### Survey findings

Reported facilitators to care-seeking are reported in Fig. 2. Overall, there were no mean differences in facilitators between women and men (*M*_women_ = 3.52, *SD* = 0.89; *M*_men_ = 3.41, *SD* = 0.59, *p* = 0.29). Looking at the individual items, in general, women tended to report quite a bit/very much agreement with most facilitators. However, less than 40% of women agreed quite a bit/very much with statements that it would be easy to find time to see a provider or that they would receive good professional care within the military or VA, while nearly 50% of men tended to agree with these statements.

#### Interview findings

Women also reported positive experiences with seeking behavioral health treatment. Some did not feel their treatment quality or access was negatively impacted by their sex or gender. Others reported an increased satisfaction with VA healthcare services in recent years as a result of structural changes benefitting women’s healthcare, as some locations had more female providers, women-only group therapy, and services related to women’s issues (e.g., MST). One participant mentioned enhanced access to childcare could help facilitate attending appointments.

## Discussion

Results provide current insight into the barriers and facilitators women veterans face when deciding whether to pursue treatment for behavioral health concerns. Although women in this study endorsed higher treatment usage and fewer barriers on survey items than men, women still reported low treatment-seeking rates relative to reported behavioral health symptomology. Interviews illuminated barriers and facilitators for women that existing veteran-specific measures may miss. The items in the barriers questionnaire used in this study^39^ could be more related to themes around masculinity (e.g., “I would be seen as weak”), and therefore of less concern to women veterans than men. In interviews, women did report many other barriers to treatment consistent with prior work^2, 4, 46^ that were perhaps not captured by the measure this study used, such as stranger harassment in VA waiting rooms or lack of women-centered care options. Women in the sample also did not endorse certain key facilitators to receiving behavioral healthcare as often as men, including being able to find adequate providers at the VA. Thus, when assessing treatment utilization, future work should consider items or questionnaires that more adequately capture the experiences of women.

Many VAs do now provide sub-clinics and behavioral health centered to women; this work illuminates that women continue to perceive barriers to care in the present day despite this. To enhance reach, a number of actions may be taken by VA providers. With women’s behavioral health services being more widely implemented, it may be that these need to be more widely promoted. VA clinicians may also benefit from continued trainings related to providing women-centered care or alleviating discomfort with stigma and discrimination in VA settings, in addition to increasing presence of women-centered programs at smaller VA centers or clinics. Treatment outside of VA settings was relatively common among the women in our sample as well, with 39.8% indicating non-VA behavioral health treatment within the past 6 months. In-person appointments were more frequently endorsed among women in non-VA settings, perhaps due to the timing of when the survey was conducted, which was a time when the VA moved primarily to telehealth sessions, but non-VA private providers may have still been having in-person sessions due to fewer restrictions. While it also may have been the case that women were receiving more frequent appointments outside the VA due to availability or comfort with non-VA settings, we are not able to conclude that from the data we have.

### Limitations and future directions

Though this study highlights the experiences of women veterans, a group whose needs remain under-met,^8^ the sample was limited as it consisted of only cisgender and mostly White women. While outside the scope of the current study, future work should examine disparities in healthcare access for other demographic subgroups, including racial/ethnic minority veterans, especially as diversity is projected to continue increasing among service members and veterans.^7, 47^ Women veterans in particular are also more likely than veteran men to hold multiple minoritized identities,^47^ suggesting that focus on intersecting minoritized identities is also needed.

Furthermore, this study was unable to capture qualitative data from women veterans with probable AUD as no participant in the interview sample met criteria for AUD (compared to 16% of the women in the survey sample). This is an important subset of the veteran population; a 2022 study reported that nearly 41% of post-9/11 veterans have met criteria for AUD in their lifetime.^48^ Similarly, about 37% of women in the full sample met criteria for mild to severe AUD within their lifetime. Future work can expand upon these findings by including the voices of women seeking care for alcohol or other substance use.

Finally, this study was conducted during the COVID-19 pandemic, which may have posed as a unique period of stressors for veterans in the sample. Though these data are important to capture, especially in light of behavioral health treatment changes (i.e., telehealth) from COVID-19, it is possible that there were stressors specific to COVID-19 contributing to our findings that our study did not capture. Thus, it remains important for future work to monitor behavioral health and treatment engagement among women veterans as time progresses.

## Implications for Behavioral Health

Though findings are consistent with existing work on barriers to care for women veterans,^21, 46^ they are important to discuss and note their replication in the context of recent changes in VA healthcare—including the aforementioned efforts to bolster care options specific to women,^20^ or namely the rise in telehealth services during COVID-19.^23–25, 49^ Telehealth has shown promise for increasing accessibility and facilitating recovery from behavioral health problems among veterans.^50–52^ Some barriers discussed by women in the sample were specific to in-person experiences, such as stranger harassment, which is reported as one of the most salient barriers to treatment seeking in the VA for women veterans.^17^ Telehealth is likely to extend beyond COVID-19 and may thus be one method of providing safe care for women within VA systems, though there remain challenges to overcome. For example, there is variation in the implementation of women-specific care across VA sites, meaning services that cater to women’s behavioral health needs may only be available for some.^53^ Indeed, recent reports indicate only ~ 30% of women veterans seeking care through the VA had access through women’s clinics.^8, 21^ Widespread implementation of women-focused and trauma-informed care is needed, and as aforementioned, getting more women veterans into care also remains of high priority.

While telehealth may be promising, some veterans indicated dissatisfaction with these services. Women in the sample reported mixed opinions on telehealth care both in qualitative and quantitative responses, with some preferring telehealth due to ease of access, and others feeling like in-person options could better meet their needs. This is consistent with prior work reporting veterans’ mixed preferences for behavioral telehealth care.^54^ Care systems thus might best serve veterans by leveraging the variety of treatment modalities available.^55^

Though challenges specific to women veterans persist, not all women in the sample had negative experiences with behavioral healthcare related to their sex or gender. Some women discussed benefitting from recent changes toward comprehensive care for women at the VA, including greater access to women providers and women-only group care settings. Further adoption of efficacious, women-focused treatment across the entire VA system is necessary to ensure that all women veterans have access to beneficial care. Additionally, given that women are now eligible to serve in combat positions,^18^ comprehensive care may be needed for post-9/11 women veterans specifically.

Challenges for women veterans in receiving adequate behavioral healthcare and support remain as their need continues to grow. The current study draws both on survey findings and direct accounts from women veterans on recent experiences with behavioral healthcare, providing a vital perspective directly from the individuals who stand to benefit from care improvements. The recency of this study’s data allows for assessment recent changes in VA behavioral healthcare—importantly, where many participants were perhaps first introduced to telehealth—and could thus offer perspectives on treatment modality preferences. As the population of women veterans grows, the current study can inform VA efforts to improve care for this historically underserved group as behavioral healthcare continues to evolve.

## Data Availability

Data may be made available upon request to Eric R. Pedersen: erpeders@usc.edu.
